# Pre-emptive purse-string suture for full-thickness resection of a gastrointestinal stromal tumor in the duodenal bulb: a case report

**DOI:** 10.1055/a-2738-7191

**Published:** 2025-11-27

**Authors:** Ying Peng, Zhenyu Wu, Chuanfang Chen, Yubo Ren, Xiaofeng Feng

**Affiliations:** 1 388288 Department of Gastroenterology, The First Affiliated Hospital (Southwest Hospital), Third Military Medical University (Army Medical University), Chongqing, China

A 56-year-old woman was admitted to our hospital with abdominal pain. Gastroendoscopy revealed a 1.5 cm × 1.4 cm submucosal tumor in the duodenal bulb ( Fig. 1 **a** ). Abdominal computed tomography showed a 1.5 cm hypervascular lesion with homogeneous arterial enhancement. Endoscopic ultrasonography indicated that the duodenal tumor originated from the fourth layer with both intraluminal and extraluminal growth, presenting a slightly hypoechoic structure ( Fig. 1 **b** ). After a preoperative team discussion and obtaining informed consent from the patient, endoscopic full-thickness resection (EFTR) was performed for tumor removal.

**Fig. 1:**
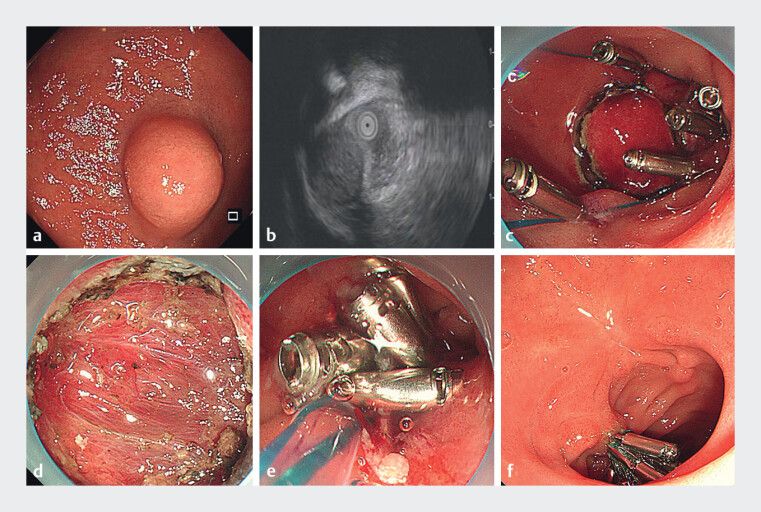
Endoscopic visualization. **a** A 1.5 cm × 1.4 cm submucosal lesion located in the duodenal bulb. **b** Endoscopic ultrasonography demonstrating a duodenal tumor arising from the fourth layer, with a slightly hypoechoic texture. **c** Pre-emptive closure of an anticipated full-thickness defect. **d** Full-thickness defect following endoscopic resection. **e** Purse-string suture closure of the duodenal bulb defect. **f** Healed scar at the duodenal bulb after 3 months.

Following submucosal injection, a circumferential incision was performed around the lesion. One metallic clip was applied for traction during the procedure. Notably, prior to complete excision of the lesion, we performed pre-emptive closure of the anticipated full-thickness defect using titanium clips combined with nylon sutures ( Fig. 1 **c–e** and Video 1 ). Histopathological examination confirmed a very low-risk gastrointestinal stromal tumor (GIST).

Use of pre-emptive purse-string suture for secure closure of full-thickness defects after endoscopic full-thickness resection of a duodenal bulb gastrointestinal stromal tumor.Video 1

The patient was discharged on postoperative day 10 without perioperative complications such as hemorrhage, perforation, or intra-abdominal infection. Follow-up gastroscopy at 3 months demonstrated complete mucosal healing at the EFTR site, with smooth scar formation and stable retention of titanium clips and nylon sutures ( Fig. 1 **f** ).

GIST can be managed via EFTR 1 . However, defect closure following EFTR particularly within the duodenal bulb remains technically challenging due to its complex anatomical location. Previous studies have reported different techniques to closing the duodenal EFTR defects 2 3 . In this case, we implemented a pre-emptive string suture technique, which successfully facilitated defect closure and significantly shortened the perforation time. This approach may offer a potential strategy for enhancing the safety and efficacy of similar endoscopic resections in the duodenum. (Written informed consent for publication of clinical images was obtained from the patient).

Endoscopy_UCTN_Code_TTT_1AO_2AO

## Notes

Endoscopy E-Videos https://eref.thieme.de/e-videos
*E-Videos* is an open access online section of the journal *Endoscopy* , reporting on interesting cases and new techniques in gastroenterological endoscopy. All papers include a high-quality video and are published with a Creative Commons CC-BY license. Endoscopy E-Videos qualify for HINARI discounts and waivers and eligibility is automatically checked during the submission process. We grant 100% waivers to articles whose corresponding authors are based in Group A countries and 50% waivers to those who are based in Group B countries as classified by Research4Life (see: https://www.research4life.org/access/eligibility/ ). This section has its own submission website at https://mc.manuscriptcentral.com/e-videos .
